# Comparative analysis of chloroplast genomes in three Araceae species: genomic difference, genetic distance and species morphology association

**DOI:** 10.3389/fgene.2025.1496262

**Published:** 2025-04-08

**Authors:** Wengang Li, Jingru Liu, Siqin Wang, Ying Ma, Lulu Cui, Yingxian Yao, Ke Sun, Lili Luo

**Affiliations:** ^1^ Department of Pharmacy, Anhui College of Traditional Chinese Medicine, Wuhu, Anhui, China; ^2^ College of Life Sciences, Anhui Normal University, Wuhu, Anhui, China

**Keywords:** araceae, genetic distance, chloroplast genomes, morphology, species morphology

## Abstract

Many species in the Araceae have extremely high medicinal value, while the chloroplast genome is relatively conserved, and the encoded and expressed bioactive substances are also abundant. Therefore, chloroplast genomes can serve as one of the basis for species evolution and are extremely important for individual material accumulation. To study the relationship between the chloroplast genome and morphology of target species, this study selected three Araceae species for chloroplast genome sequencing assembly, downloaded the complete chloroplast genomes sequences of another 11 Araceae species. Grouping based on genetic distance, we analyze the association between chloroplast genome structure and morphology. The results showed that there were significant differences in genome size among the three species, but Relative Synonymous Codon Usage (RSCU) exhibited high similarity; Based on the phylogenetic tree, these 14 species can be divided into three branches, with differences in genes such as *rrn4*, *rrn5*, *rrn23*, and *trnN* among species within each branch; Morphologically, the length of the male inflorescence in BranchⅢ is significantly greater than that in BranchⅡ; There is a strong positive correlation between the length of the plant stem and the three parameters (Length of LSC, Length of SSC and Length of chloroplast genome) of the genome. This study conducted correlation research from the perspective of chloroplast genome and species morphology. On the one hand, the genetic distance and chloroplast genome structure differences between the target species were determined, and on the other hand, explored the correlation between chloroplast genome and species morphology, providing a theoretical basis for the study of phylogenetic relationships and morphology of Araceae species.

## 1 Introduction

### 1.1 Chloroplast genome structure and function

Chloroplasts are key organelles for photosynthesis, with the highest abundance of soluble proteins, membrane proteins, pigments, and lipids (Kirchhoff, 2019). The chloroplast genome size of most land plants is 120–160 kb (Wicke et al., 2011), containing inverted repeats (IR), a large single copy (LSC) region of 80–90 kb, and a small single copy (SSC) region of 10–20 kb (Wang and Lanfear, 2019). The IR region is highly conserved among different plants, with a length ranging from 20,000 to 25,000 bp (Morley et al., 2019), and within the IR region, intronic sequences are also considered to be highly conserved (Daniell et al., 2016). In most land plant individuals, two chloroplast structural haplotypes occur at equal frequencies. However, those species whose chloroplast genomes lack inverted repeats or have short inverted repeats have only one structural haplotype (Wang and Lanfear, 2019). Chloroplasts can dynamically adjust their energy conversion and metabolic performance according to plant metabolism and environmental demands. In this regard, they have developed different mechanisms to minimize and deal with toxic photochemical side reactions (reactive oxygen species, ROS), which are the inevitable cost of converting sunlight in an oxygenated atmosphere (Kirchhoff, 2019). In many angiosperms, cpDNA has a multibranched linear structure (Mower and Vickrey, 2018). However, the amount of cpDNA varies greatly during plant development (Oldenburg and Bendich, 2015). During evolution, many ancestral chloroplast genes have been transferred from cpDNA to the nucleus, a process known as endosymbiotic gene transfer (Ku et al., 2015).

### 1.2 Medicinal value of araceae plants

Many species of the Araceae have high medicinal value. *Pinellia pedatisecta* (distributed in central and southern China, mainly growing in forests, valleys, and shaded areas (Abdullah et al., 2021).) has the effects of reducing phlegm, relieving nausea, dissolving lumps, and dissolving tumors. Recent pharmacological studies have shown that *P. pedatisecta* may be an effective anticancer immunomodulatory drug (Wang et al., 2021), *P. pedatisecta* contains heptacosterol, quercetin, sitosterol, and stigmasterol, which interact with the core targets AKT1, MAPK3, and ESR1. It has strong binding activity and can be used as an effective drug for the treatment of melanoma through the PI3K/Akt pathway (Wang et al., 2022). *Arisaema erubescens* (Widely distributed in southern China and Southeast Asian countries.) is a perennial herbaceous plant of the Araceae. In medicine, it is used as an important detoxifier for the treatment of several biological diseases (Li, 2007), Its main effects are to remove dampness and resolve phlegm, prevent convulsions, and promote the reduction of sclerosis and swelling (Yang et al., 2007), Its extracts show anticancer properties (Ducki et al., 1996). It has anticoagulant, gastric analgesic, sedative, antiemetic, anti-inflammatory, and antitumor activities (Dong et al., 2015). *Arisaema heterophyllum* [distributed in central and western tropical Africa, Angola. Growing on rocky ground along riverbanks or rivers, as well as in shaded areas of forests (Abdullah, et al., 2021)] rhizomes or tubers are used in traditional Chinese medicine to treat cancer and depression and relieve pain (Roshan et al., 2017). Studies have shown that *A. heterophyllum* extract can induce cell apoptosis and autophagy by inhibiting the PI3K/Akt pathway (Li-Xing et al., 2016).

As a relatively independent organelle, chloroplast genome plays an extremely important role in energy and material accumulation. Many species of Araceae have extremely high medicinal value. Therefore, this study extracted and sequenced the chloroplast genomes of three Araceae species, used the genetic distance between the three Araceae species as the basis for grouping, analyzed the correlation between phenotypic parameters and chloroplast genome parameters, and provided molecular evidence for the evolution and variation of Araceae species.

## 2 Materials and methods

### 2.1 Sample collection and DNA extraction

Fresh healthy leaf of three plants of the Araceae (*Pinellia pedatisecta*, *A. heterophyllum* and *A. erubescens*) were collected from the greenhouse of Anhui College of Traditional Chinese Medicine in Wuhu. Cetyl trimethyl ammonium bromide (CTAB) were used to extract whole-genomic DNA from 100 mg of fresh leaf tissue. Elute each extracted DNA in 125 µL of elution buffer. DNA concentration and quality were determined using 1% gel electrophoresis and Nanodrop (ThermoScientific, Delaware, United States).

### 2.2 Library construction

We used 55 μL (20 ng/μL) of DNA for library preparation based on the protocol of Illumina TruSeq kits in the Pires lab at the University of Missouri, Columbia following the TruSeq DNA Sample Preparation Guide protocol (Illumina, Inc., 2010), except where noted. The DNA was sheared by sonication of 15–24 min using a Bioruptor (Diagenode, Inc., New Jersey, United States). X-tracta disposable gel extraction tools United States Scientific, Ocala, Florida, United States) were used to perform gel extractions for size-selection of samples between 200–400 bp. The gel extractions were purified with the Gel Extraction kit (Qiagen) for the end repair, adenylation of 3′ ends, ligation, and enrichment steps. The sheared DNA was visualised for size selection by running on 1% gel for 1 h at 120 V. Before sequencing, we first used ABI 3730xl DNA analyzer to determine the number and size of DNA fragments. Only qualified samples were used for the next step of sequencing. Qualified DNA libraries were sequenced using Illumina HiSeq 2000 (Illumina, Inc., San Diego, California) with a single-end read length of 100 bp. After sequencing, the read quality of each sequenced sample was assessed using FastQC (Andrews, 2017), and then RawData was output using MultiQC (Ewels et al., 2016) for subsequent assembly.

### 2.3 Genome assembly and annotation

We used the default parameters of Fast-Plast v. 1.2.2 (McKain and Wilson, 2017) and NOVOPlasty (Dierckxsens et al., 2017) for chloroplast assembly. In this process, Trimmomatic v. 0.36 (Bolger et al., 2014)was first used to trim the adapters and quality of the original Illumina reads, and then Bowtie2 v. 2.2.9 (Langmead and Salzberg, 2012) was used to adjust the parameters to “very-sensitivelocal” to trim the trimmed reads. Mapping with GenBank. Mapped reads were assembled using SPAdes v. 3.9.0 (Bankevich et al., 2012) and K - mers were assembled by selecting the “assembler only” option based on the size of the reads. Chloroplast genomes, in circular form, were annotated using GeSeq with BLAT search of 85% identify for annotation of proteincoding genes, rRNAs and tRNAs. The annotations of tRNAs were further confirmed by using tRNAscan-SE v. 2.0.3 (Lowe and Chan, 2016) and ARAGORN v. 2.38 (Laslett and Canback, 2004) by selection of default parameters of chloroplast genome. Blast was used to search for homologous genes in the chloroplast genome to further confirm the positions of the start codon and stop codon. Finally, using Geneious Prime to generate a table of annotated genes, submit the sequencing results and annotation results to NCBI (National Center for Biotechnology Information), and then using Geneious Prime to create a fully annotated chloroplast circle diagram (Lohse et al., 2007).

### 2.4 Data collection and comparison between plant chloroplast genomes

To compare the genomic features of the three Araceae species, we used Geneious R8.1 to determine codon bias and amino acid usage frequency (Kearse, et al., 2012). IRscope was used to visualize the contraction and expansion of IR regions at chloroplast genome junctions (Amiryousefi, et al., 2018), mVISIT was used to focus on gene rearrangements (Darling et al., 2004), and we obtained the genome sequences from NCBI. Complete chloroplasts of 11 other Araceae species [*Arisaema amurense* (NC085264), *Arisaema franchetianum* (MN046885), *Arisaema flavum* (NC062735), *Arisaema ringens* (NC044118), *Arisaema decipiens* (NC064687), *Arisaema nepenthoides* (MW338731), *Arisaema bockii* (MZ380241), *Arisaema prazeri* (NC072165), *P. peltata* (NC052862), *P. ternata* (NC027681), *P. cordata* (MT863558)], and a orchid species as an outgroup [*Bletilla sinensis* (ON243844)] were downloaded from NCBI. A phylogenetic tree was then constructed using iqtree (1,000 iterations, 1,000 replicates) with the Neighbor-Joining (NJ) Algorithm (Nguyen et al., 2015). In order to compare the phenotypic characteristics of Araceae plants, we collected six indicators including tuber diameter, petiole length, inflorescence stalk length, tube diameter, female inflorescence length and male inflorescence length according to Flora of China (Li, 2007) to analyze the phenotypic characteristics of plants and grouped them according to their evolutionary relationships. Using the Psych package to calculate the correlation coefficient between genome size and plant phenotype, and drawing the correlation heatmap by ComplexHeatmap. Each block is represented by a different color to indicate different correlations, and coefficients are marked within the block. Significance is represented by *, where * represents 0.01 < *P* < 0.05, and not significace with *P* > 0.05.

## 3 Results

### 3.1 Comparison of genomes after species sequencing and assembly

**FIGURE 1:**
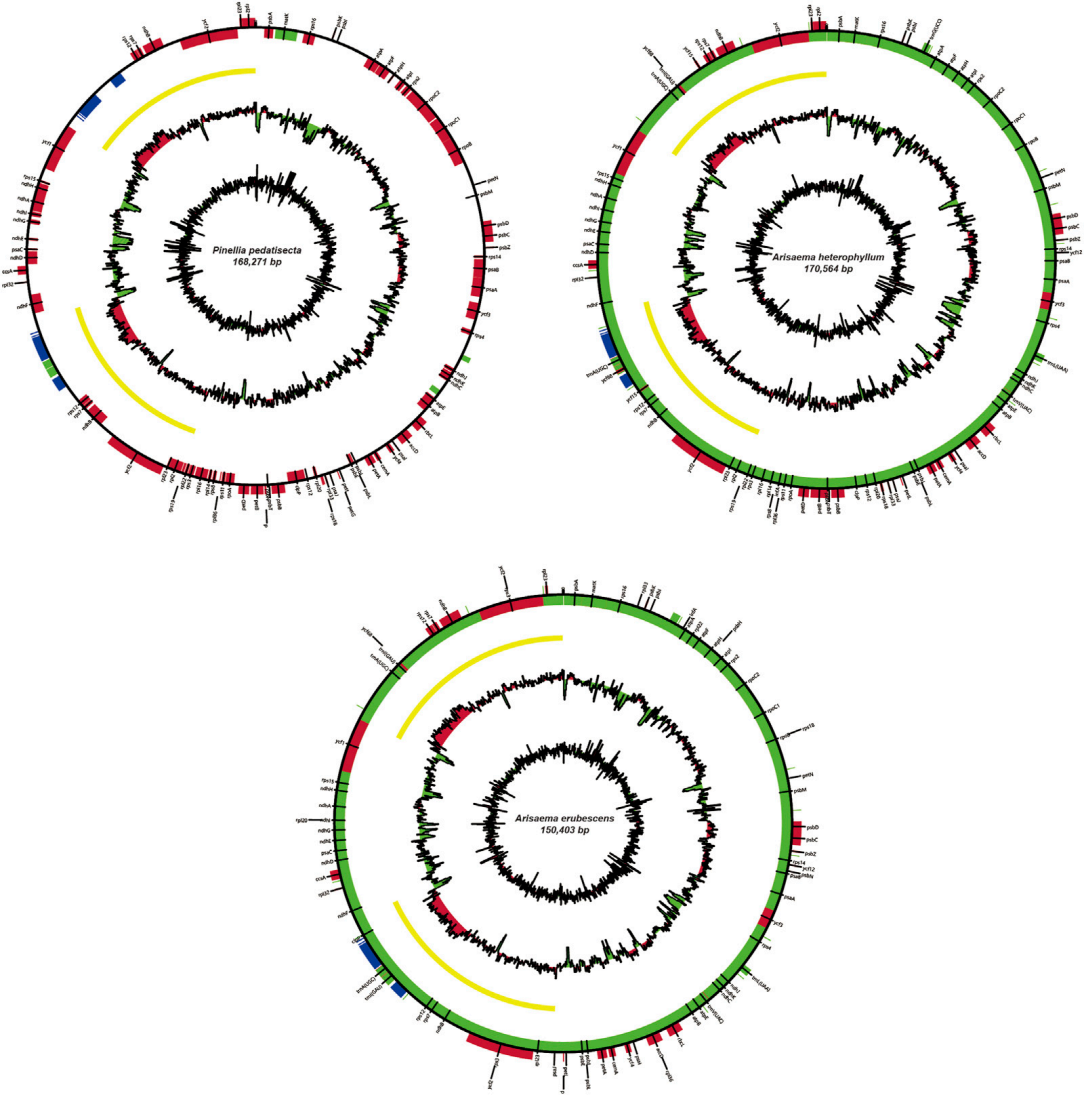
Schematic map of overall features of the chloroplast genome of *Arisaema heterophyllum*, *Arisaema erubescens* and *P. pedatisecta*. The map contains four circles. From the center going outward, the first circle shows the GC skew (green means less than 0, red means greater than 0). The next circle shows the GC content (red represents greater than the average GC content of the genome, and green represents less than the average GC content of the genome). The third circle shows the inverted repeat sequence. The fourth circle shows the gene location (mRNA is red, tRNA is green, rRNA is blue).

**FIGURE 2:**
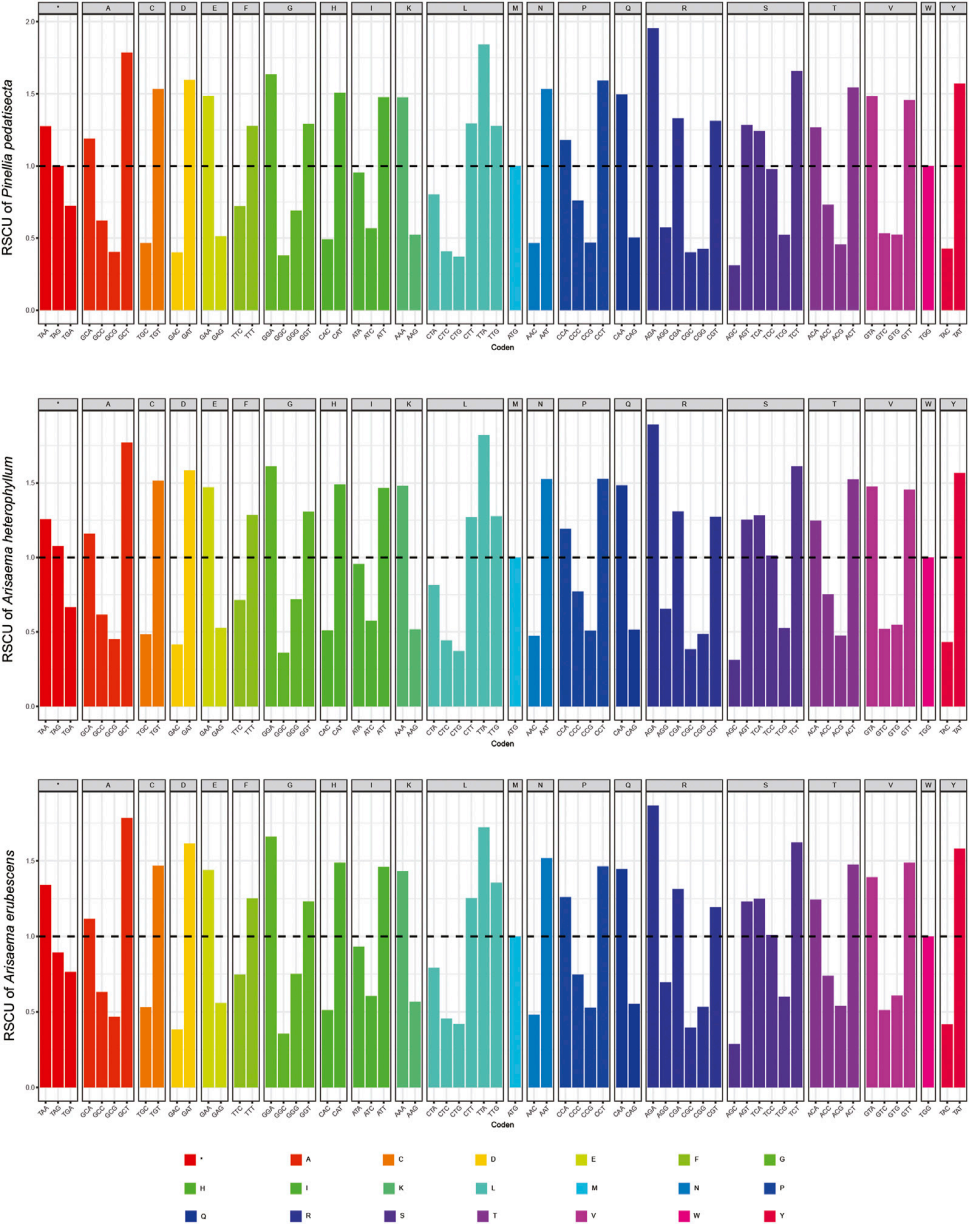
Statistical comparison of relative synonymous codon usage (RSCU) of chloroplast genes in three species of araceae.

The chloroplast genome sizes of *P. pedatisecta*, *A. heterophyllum*, and *A. erubescens* differed greatly (168,262 bp, 170,584 bp, and 150,394 bp, respectively) (Figure 1). Table 1 shows that there were great differences in the total number of genes obtained from the chloroplast genomes of the three species. The lowest total number of genes was 98 (*P. pedatisecta*) and the highest was 147 (*A. heterophyllum*). However, the number of rRNA genes in the three species was 8. The number of tRNAs in both plants was the same, which was much larger than that in *P. pedatisecta*. The protein-coding genes in *P. pedatisecta* were the highest, at 99, while *A. erubescens* had the lowest, at only 83 genes. In addition, the GC content of the three plants was also very similar (42% (*P. pedatisecta*), 41% (*A. heterophyllum*), and 43% (*A. erubescens*)). Relative synonymous codon usage showed that codons with A or T rather than C or G at the 3′end had a higher coding rate. The RSCU of codons with A/T at the 3′end was mostly ≥1, while the RSCU of codons with C or G at the 3′end was mostly ≤1. Amino acid frequency analysis showed that leucine and isoleucine had the highest frequencies, while cysteine was a rare amino acid. Overall, we found that the RSCU of the three Araceae species had high similarity (Figure 2).

**TABLE 1:** Comparison of the general information of three species of Araceae.

Species names	Genome	PCGs	rRNA	tRNA	Total genes
*Pinellia pedatisecta*	168,262	85	8	5	98
*Arisaema heterophyllum*	170,584	99	8	40	147
*Arisaema erubescens*	150,394	83	8	40	131

### 3.2 Determination of phylogenetic relationships and grouping of araceae

**FIGURE 3:**
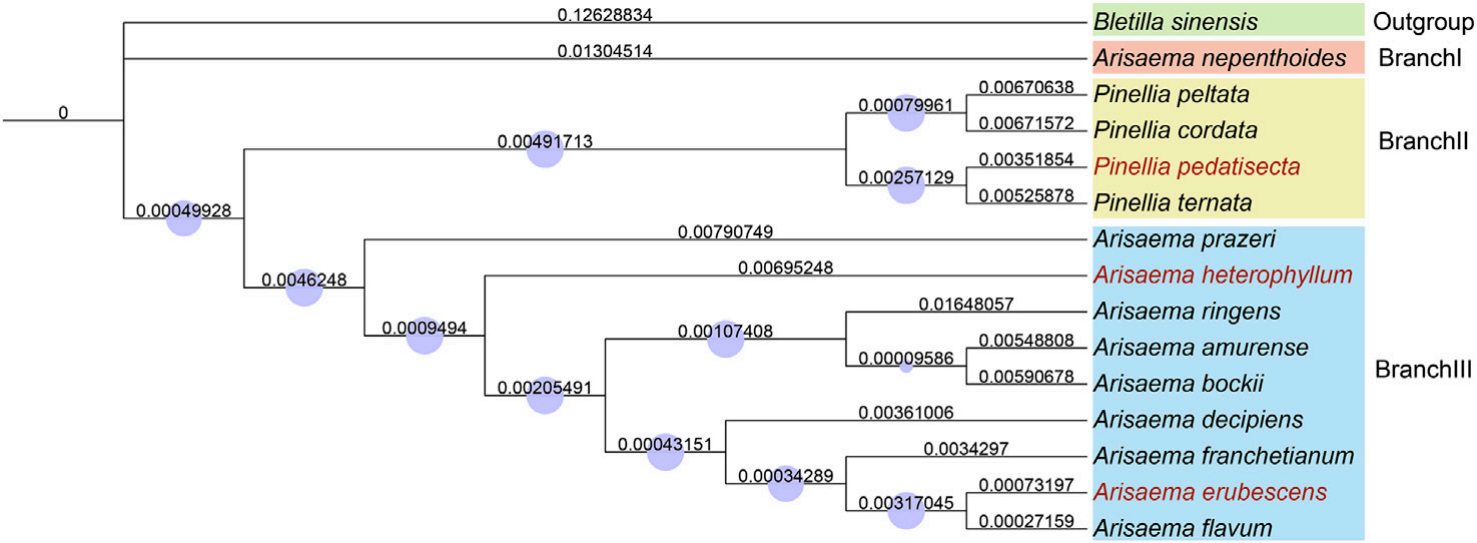
A Neighbor-Joining tree based on the entire chloroplast genome sequence of 15 species in the Araceae. The number above the branch indicates the branch length, the blue circle represents the Bootstraps, and the red font represents our sample.

To determine the evolutionary relationship of these three species in the Araceae, we selected the chloroplast whole genome sequences of 11 species from Arisaema and Pinellia, with *B. sinensis* as the outgroup, and constructed a phylogenetic tree based on the NJ Algorithm (Figure 3). The results indicate that *P. pedatisetta* and other species of *Pinellia* cluster on the same large branch, while *A. heterophyllum* and *A. erubescens* cluster on the same large branch as *Arisaema*. Strangely, in plant taxonomy, the *A. nepenthoides*, which belongs to *Arisaema*, forms a separate branch and seems to have significant differences in chloroplast genome variation compared to *Arisaema* species. Therefore, based on the tree diagram results, we artificially divided them into three different branches, namely, BranchⅠ (*A.nepenthoides*), BranchⅡ (*P. peltata*, *P. cordata*, *P. pedatisecta*, *P. ternata*), and BranchⅢ (*A.prazeri*, *A. heterophyllum*, *A. ringens*, *A. amurense*, *A. bockii*, *A. decipiens*, *A. franchetianum*, *A. flavum*), for the convenience of subsequent analysis.

### 3.3 Comparison of chloroplast genomes in araceae

**FIGURE 4:**
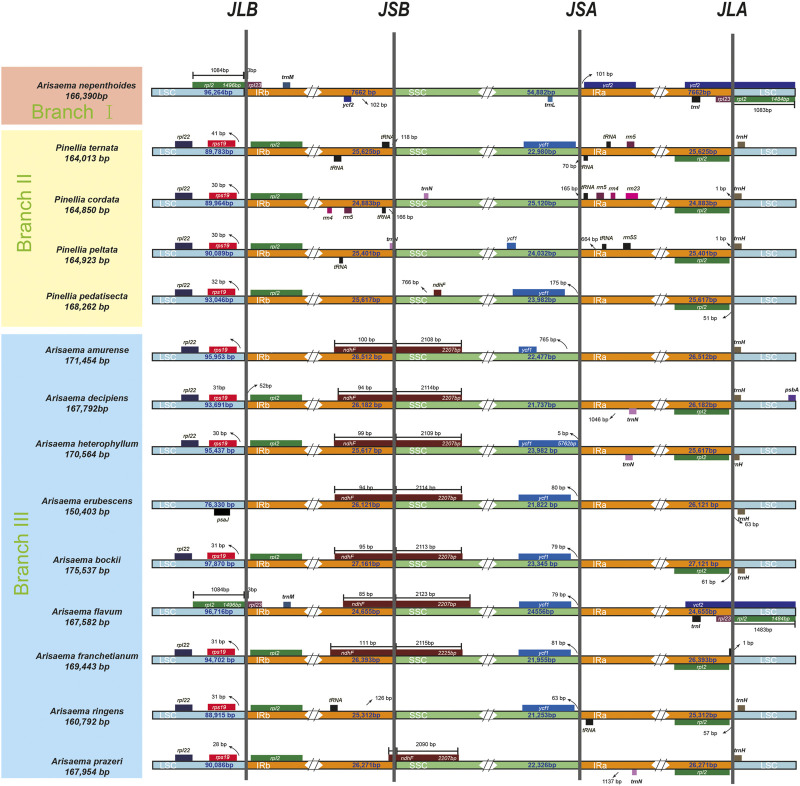
Comparisons of the borders of LSC, SSC, and IR regions among Araceae chloroplast genomes.

In order to analyze the length differences and gene location differences in different regions, we used the IRscope online tool to analyze the IR contraction and expansion phenomena in the chloroplast genomes of different branching species (Figure 4). Divide the 14 species into three branches based on the results of the tree diagram. BranchⅠ contains one species (*A. nepenthoides*) with a chloroplast genomes length of 166,390 bp. *A. nepenthoides* lacks the *rpl22* and *rps19* genes in the LSC region, while the majority of *rpl23* regions have LSC regions and lack *ndhF* and *trnl* genes. BranchⅡ contains four species, with chloroplast genomes lengths ranging from 164013bp to 168262bp. Compared with the other three species, *P. cordata* contains more small gene, such as *rrn4*, *rrn5*, *rrn23*, *trnN.* Only *A. prazeri* contains the *ndhF* gene, spanning the IR and SSC regions. BranchⅢ contains eight species, with chloroplast genomes lengths ranging from 150,403 bp to 75,537 bp. Compared to the other seven species, chloroplast genomes of *A. ringens* do not contain the *ndhF* gene, *A. erubescens* does not contain the *rpl2* gene, and only *A. flavum* contains the *ycf2* gene.

### 3.4 Analysis of phenotypic parameters of araceae plants

**FIGURE 5:**
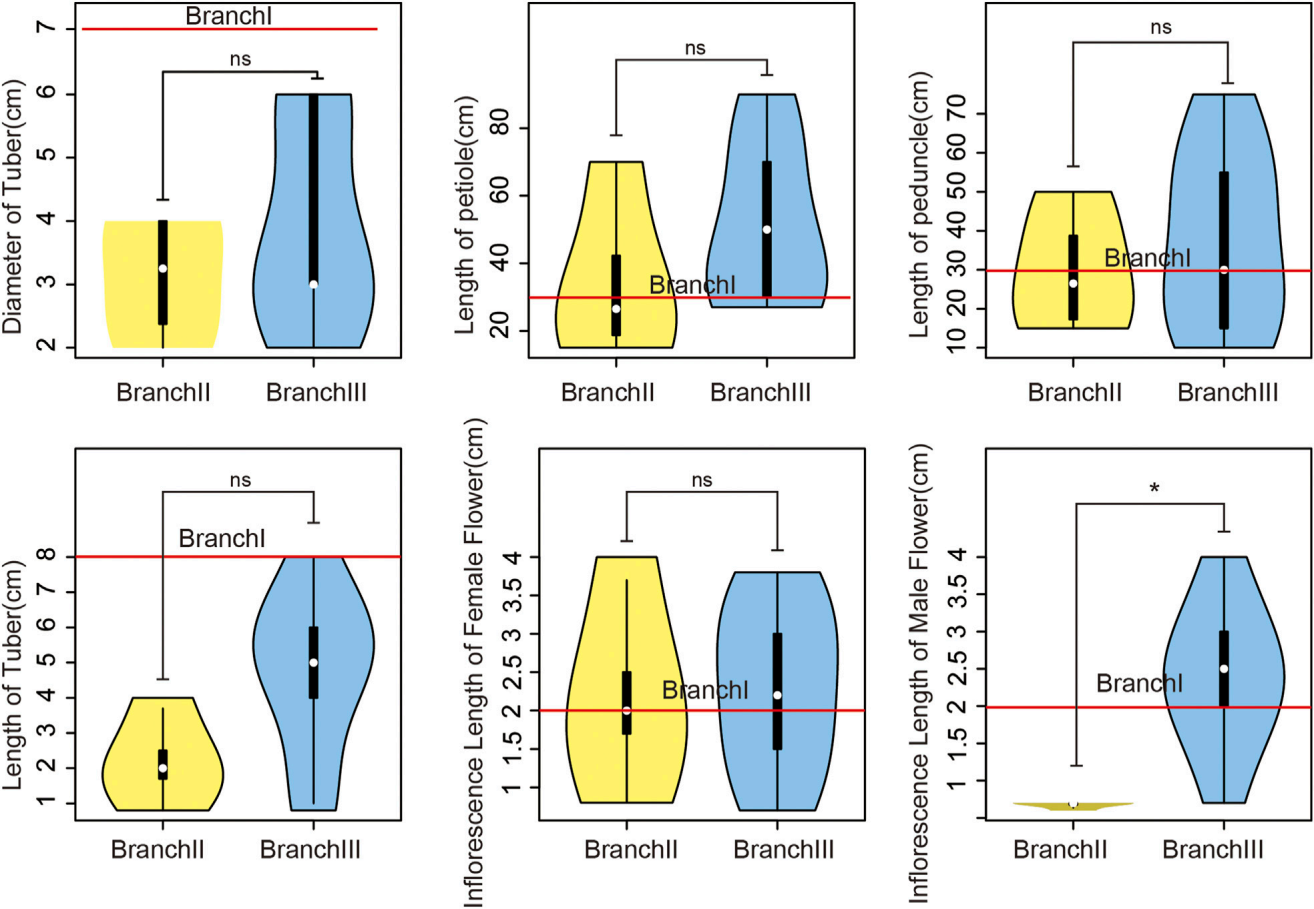
Morphological differences between BranchⅡ with BranchⅢ and red line is BranchⅠ. The horizontal axis shows the branch while the vertical axis denotes the morphological data. The white point represents the mean value, the outer contour represents sample distribution, the upper side of the black box in the middle represents the upper 1/4, and the lower side represents the lower 1/4. ns (*P* > 0.05), * (0.01 < *P* < 0.05).

In order to analyze the differences in plant morphology of different branches, we collected six indicators, including tuber diameter, petiole length, inflorescence stalk length, tube diameter, female inflorescence length and male inflorescence length, based on the records of 14 species in Flora of China. The results showed (Figure 5) that the tuber diameter of the 14 species ranged from 2 to 7 cm, the petiole length ranged from 15 to 90 cm, the inflorescence stalk length ranged from 10 to 60 cm, the tube length ranged from 0.8 to 8 cm, the female inflorescence length ranged from 0.5 to 3.8 cm, and the male inflorescence length ranged from 0.6 to 4 cm. The male inflorescence length of BranchⅢ is significantly higher than that of BranchⅡ, but there is no significant difference in the other five morphological parameters between branches.

### 3.5 Analysis of association between plant phenotype and chloroplast genome

**FIGURE 6:**
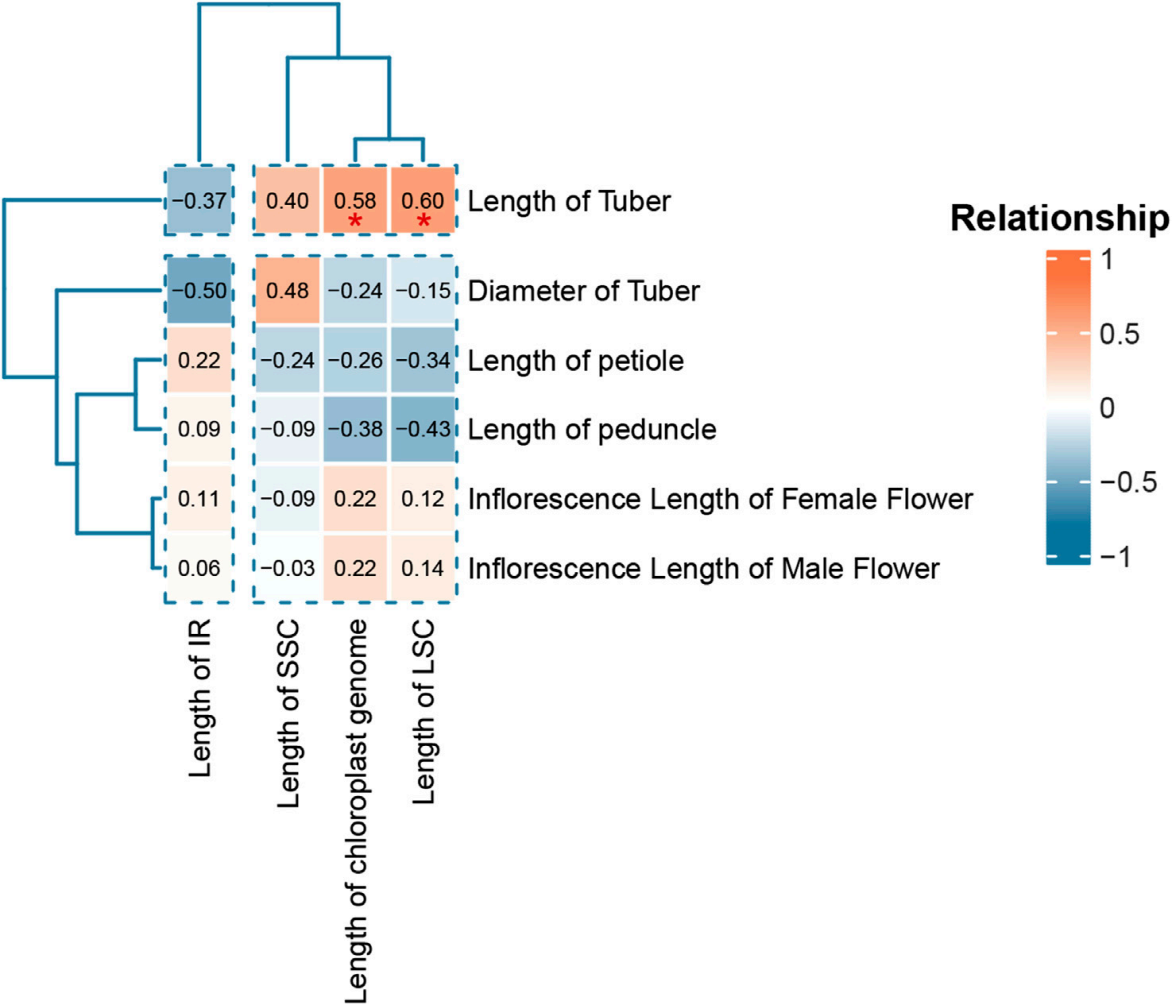
Heat map of the correlation between chloroplast genome parameters and morphological data. The correlation calculation method is Pearson’s correlation coefficient.* (0.01 < P < 0.05), and Not labeled indicates not significance (*P* > 0.05).

To analyze the correlation between chloroplast genome and species phenotypic data, we calculated the correlation coefficients between chloroplast genome parameters (full length, LSC length, SSC length and IR length) and plant phenotypes (tuber diameter, petiole length, inflorescence stalk length, tube diameter, female inflorescence length and male inflorescence length) and displayed them in a heat map (Figure 6). The results showed that plant tube length showed a strong positive correlation with three genome parameters, namely LSC length (R = 0.6), chloroplast genome length (R = 0.58) and SSC length (R = 0.4). SSC length showed a strong positive correlation with tube diameter (R = 0.48). IR length showed a strong negative correlation with tube length (R = −0.37) and tuber diameter (R = −0.5). LSC length showed a strong correlation with petiole length (R = −0.34) and inflorescence stalk length (R = −0.43). The full length of chloroplast genes also showed a strong correlation with petiole length (R = −0.326) and inflorescence stalk length (R = −0.38).

## 4 Discussion

### 4.1 Chloroplast gene loss and changes in species survival strategies

In this study, Illumina sequencing technology was used to assemble the chloroplast genomes of three Araceae species and conduct comparative analysis. All methods were carried out in accordance with relevant guidelines and regulations. According to the results of the phylogenetic tree, we believe that among the branches divided, the one with the largest difference in chloroplast genome structure is *A. nepenthoides*, the one with the largest difference in BranchⅡ is *A. prazeri*, and the one with the largest difference in BranchⅢ is *A. ringens*. In BranchⅢ, the main difference between *A. nepenthoides* and the other three species is the loss of *rpl22* gene and *rps19* gene. In plants, the transfer of plastid genes to nuclear genes is a very common phenomenon, such as *infA* (Millen et al., 2001) of rosids, *rpl22* (Gantt et al., 1991) of Pisum, *rpl32* (Cusack and Wolfe, 2007) of some Salicaceae, and *rpoA* (Sugiura et al., 2003) of mosses. The loss of *rpl22* gene in *A. nepenthoides* may be due to transfer to nuclear genes. However, there is no literature showing that the transfer of this gene has any effect on the evolution and functional expression of this species. The *ndh* genes are homologous to components encoding mitochondrial and bacterial respiratory complex I (NADH dehydrogenase) (Ohyama et al., 1986; Shinozaki et al., 1986). Chloroplasts are evolutionarily derived from primitive endosymbionts in host cells, cyanobacteria (Martin et al., 2002), Many genes from cyanobacteria were gradually transferred to the nucleus of the host cell, and the engulfed cyanobacteria evolved into only partially autonomous chloroplasts. Most chloroplast proteins are encoded in nuclear DNA, and only a few chloroplast proteins (about 100) are encoded in genes retained in plastid DNA; among them are ndh genes. Although these genes have been lost in most algal lineages, they are conserved in the plastid DNA of Streptophyta and derived land plants. This suggests that *ndh* genes have some advantages in the adaptation from aquatic to terrestrial environments (Sabater, 2021). Angiosperms that have lost their plastid *ndh* genes survive in mild and moderate stress environments or adopt heterotrophic or carnivorous metabolism to compensate for their low or absent photosynthetic efficiency. As biological evolution changes over time, different environments put angiosperms without *ndh* at risk of permanent extinction, just as the endangered golden plants may have done (Sun et al., 2020). In this study, many species in the *Pinellia* lacked the *ndhf* gene, and a few species in the *Araceae* lacked the *ndfh* gene. The lack of *ndf* genes can lead to differences in the way plants survive, which may indicate that the species of the *Pinellia* and *Araceae* have different living environments, and this difference is determined by genes. Araceae has higher photosynthesis efficiency than species in the *Pinellia*.

### 4.2 Relationship between genome structure and plant phenotypic differences

Genome size has important implications for the adaptive evolution of species, which influence phenotype through gene expression (Bennett and Smith, 1976) and may further influence cross-compatibility between genotypes. In plants, DNA amount can be related to several characteristics such as minimum generation time and ecological behavior (Eaton et al., 2004). In current research, people pay more attention to the correlation between nuclear gene expression and plant phenotype, while there are relatively few studies on the species phenotype of chloroplast genome. Studies have shown that there are differences in growth habits and leaf color between wild-type and cultivated individuals of *Poa pratensis*, and there are also significant differences in chloroplast genome length (Raggi et al., 2015). *Buckwheat* species differ mainly in height, leaf shape, seeds and sowing type. There are differences in chloroplast genome length and number of SSRs between these four species (Wang et al., 2017); differences in morphological characteristics such as style, stigma, stigma, male scales, seed shape and integument decoration of *Cuscuta* reproductive organs, chloroplast genome analysis results showed significant differences in chloroplast genome length and gene order between the two plants (Park et al., 2019). This study collected 6 phenotypic data including tuber diameter and petiole length of 14 species of Araceae in Flora of China, and conducted correlation analysis with chloroplast genome parameters. The data showed that there is a strong relationship between chloroplast gene parameters and plant phenotype. Although this study does not provide direct evidence of the correlation between chloroplast genome and plant phenotypes, it provides an idea and lays the foundation for subsequent research.

## Data Availability

The datasets presented in this study can be found in online repositories. The names of the repository/repositories and accession number(s) can be found below: https://www.ncbi.nlm.nih.gov/genbank/, No. OR772808.
